# Overcoming thermo-optical dynamics in broadband nanophotonic sensing

**DOI:** 10.1038/s41378-021-00281-y

**Published:** 2021-07-07

**Authors:** Mingkang Wang, Diego J. Perez-Morelo, Vladimir Aksyuk

**Affiliations:** 1 Microsystems and Nanotechnology Division, National Institute of Standards and Technology, Gaithersburg, MD 20899 USA; 2 Institute for Research in Electronics and Applied Physics, University of Maryland, College Park, MD 20742 USA

**Keywords:** Optical sensors, Nanophotonics and plasmonics, NEMS

## Abstract

Advances in integrated photonics open up exciting opportunities for batch-fabricated optical sensors using high-quality-factor nanophotonic cavities to achieve ultrahigh sensitivities and bandwidths. The sensitivity improves with increasing optical power; however, localized absorption and heating within a micrometer-scale mode volume prominently distorts the cavity resonances and strongly couples the sensor response to thermal dynamics, limiting the sensitivity and hindering the measurement of broadband time-dependent signals. Here, we derive a frequency-dependent photonic sensor transfer function that accounts for thermo-optical dynamics and quantitatively describes the measured broadband optomechanical signal from an integrated photonic atomic force microscopy nanomechanical probe. Using this transfer function, the probe can be operated in the high optical power, strongly thermo-optically nonlinear regime, accurately measuring low- and intermediate-frequency components of a dynamic signal while reaching a sensitivity of 0.7 fm/Hz^1/2^ at high frequencies, an improvement of ≈10× relative to the best performance in the linear regime. Counterintuitively, we discover that a higher transduction gain and sensitivity are achieved with lower quality-factor optical modes for low signal frequencies. Not limited to optomechanical transducers, the derived transfer function is generally valid for describing the small-signal dynamic responses of a broad range of technologically important photonic sensors subject to the thermo-optical effect.

## Introduction

The rapid development of integrated photonics and nanotechnology has enabled a wide range of nanophotonic sensors applicable for thermal^1,2^, magnetic^3,4^, gas^5,6^, force^7^, and displacement^8,9^ sensing. High-quality factor (*Q*) nanophotonic cavities strongly enhance local light-matter interactions via their small optical mode volumes and extended photon lifetimes, enabling an unmatched combination of ultrahigh precision and ultrawide bandwidth for optical sensing in a variety of applications. This includes cavity-optomechanical on-chip motion transduction^10,11^, where, for example, a low-loss nanomechanical resonator has been combined with a high-finesse optical cavity for ultrafast nanoscale optomechanical atomic force microscopy (AFM)^12–14^ and recently applied for the direct measurement of local chemical and thermal properties using photothermal-induced resonance (PTIR)^15^.

Generally, the signal-to-noise ratio of nanophotonic sensors improves with increasing transduction gains achieved by increasing the optical power in the photonic cavity. However, the very same beneficial qualities—small volumes and high *Q*—increase local optical heating, which quickly becomes significant at even modest input powers. This heating limits the power that can be used for better sensitivity since with a further power increase, the cavity frequency becomes power- and time-dependent due to the thermo-optical effects^16–19^, complicating the transduction dynamics and making it difficult to recover the sensor input stimulus signal from the detected photonic response for any broadband stimuli. Conventional optical-resonator-based sensors choose to either operate the sensor in the low optical power, low-gain linear broadband measurement regime, avoiding complex thermally induced transduction at low frequencies, or abandon the accurate measurement of low- and intermediate-frequency signals to pursue high transduction gain at high frequencies. Our work removes this trade-off between the gain and the detection bandwidth imposed by the thermo-optical nonlinearity. Taking the AFM application as an example, simultaneous and accurate measurements of static and dynamic probe displacements are critical to obtaining long-range and short-range sample topography during rapid scanning. They are also key to measuring and quantitatively interpreting dynamic processes at the nanoscale^15,20^. An accurate quantitative description of the optical-power-dependent sensor transfer function (the change in the photonic response per unit mechanical displacement) across the mechanical frequency range down to DC will remove the present input power limitation and push the sensitivity to the next level. Not limited to optomechanics, the enhanced thermo-optic effect is ubiquitous in nanophotonic cavity sensing, and a transfer function accounting for it would broadly enable operating this class of sensors with increased sensitivity and transduction bandwidth.

The present work aims to advance nanophotonic sensing, leveraging highly integrated optomechanical AFM probes^14,15^ as a well-established high-performance experimental platform. We derive and experimentally confirm an optical power- and frequency-dependent optomechanical transfer function working down to the low-frequency range, where at high optical power, the optomechanical transduction gain is affected by thermo-optical tuning. We first use the thermodynamic fluctuations of a nanomechanical cantilever as a broadband stimulus, measured by an evanescently coupled microdisk in the thermo-optically nonlinear regime. The derived transfer function, requiring no adjustable parameters, is found to accurately describe the photonically measured signal power spectral density over a wide range of frequencies and different degrees of thermo-optical nonlinearity. Additionally, by driving the cantilever and calibrating the displacement, we demonstrate and quantify the increased transduction gain and sensitivity in the high optical power regime. When the AFM probe operates in the thermo-optical nonlinear regime, the sensitivity is increased by ≈10 times compared to the best performance in the linear regime, reaching ≈0.7 fm/Hz^1/2^. Furthermore, by considering the effect of the optical mode *Q* on the transduction gain with thermal dynamics included, we discover and experimentally confirm that, counterintuitively, a lower *Q* cavity mode could provide a larger transduction gain and sensitivity for low-frequency signals compared to a higher *Q* mode. Understanding signal transduction in the presence of thermo-optical dynamics enables the high-power, high-sensitivity operation of a general class of photonic sensors, with the optomechanical AFM probe being just one example.

## Results and discussion

### Experimental setup

**Fig. 1:**
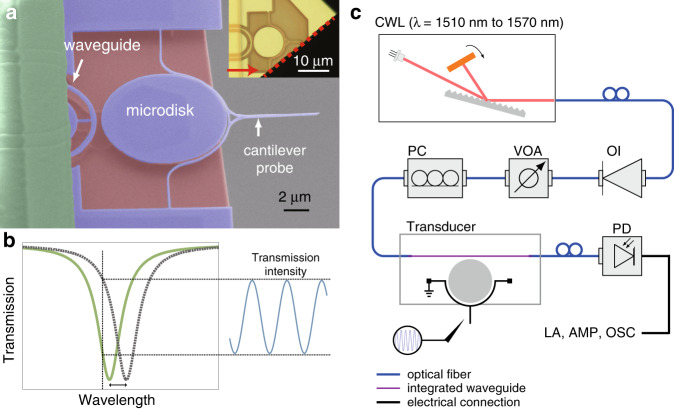
Introduction to the system. **a** Colorized scanning electron micrograph of an optomechanical AFM probe with a double-clamped cantilever. The cantilever has a nominal width of 150 nm and is separated by a gap of 200 nm from a 10 μm diameter silicon microdisk. The cantilever and silicon disk are nominally 260 nm thick. The disk is supported by a silica post underneath. An optical photo of a single-clamped device is shown in the inset. The red arrows and the dashed lines indicate the position where the cantilever is cleaved from the anchor point. **b** During the measurement, the fiber-coupled laser wavelength is fixed (vertical dashed line). The motion of the cantilever changes the cantilever-disk gap, shifting the resonances of the microdisk. At the working wavelength on the shoulder of the optical resonance, the shifting resonance generates a varying transmission intensity. **c** Schematic of the measurement setup containing a continuous-wavelength tunable diode laser (CWL), optical isolator (OI), variable optical attenuator (VOA), polarization controller (PC), electronic amplifier (AMP), lock-in amplifier (LA), oscilloscope (OSC), and photodetector (PD). The cantilever is grounded, and a metal tip drives the cantilever by applying an electrostatic force.

The nanophotonic sensor under study is an optomechanical device consisting of a curved cantilever probe held in the nearfield of a microdisk optical cavity supporting whispering-gallery modes (WGMs). An experimental demonstration of the full operation of this device as an AFM sensor has been reported^14^, and it has enabled an unprecedented new data collection regime in the AFM/PTIR setup. Figure 1a shows an electron micrograph of the optomechanical AFM probe. The cantilever has multiple mechanical modes; however, only the fundamental in-plane mode of a (5.46 ± 0.01) MHz eigenfrequency is dominant, while other mechanical modes (for both the cantilever and the microdisk itself) are either far above the fundamental mode or do not produce a strong optical readout signal due to motion being mostly orthogonal to the microdisk plane. In some experiments, we cleaved the device such that the cantilever was supported at only one end (cleaved at the red dashed line in the inset), lowering the mechanical stiffness and the fundamental in-plane mechanical mode frequency to (310 ± 0.2) kHz, which is beneficial for studying the frequency-dependent transfer function in the thermally driven case. Note that the microdisk thermo-optical nonlinearity effects are the same for both the double-clamped and single-clamped cantilever probes. Throughout the paper, the reported uncertainties are one-standard deviation statistical uncertainty, unless noted otherwise.

The integrated optical cavity works as a high-*Q* interferometric motion transducer, where the motion of the cantilever modulates the optical mode frequencies. The input laser is tuned to a shoulder of the cavity resonance such that at small optical powers where the thermo-optical effect is negligible, the variations in the transmitted optical power are proportional to the displacement of the cantilever, as shown in Fig. 1b.

Figure 1c presents the setup for measuring the optical and mechanical responses of the system. Transmission measurements are carried out by using a continuous-wavelength tunable laser (1510–1570 nm) as the light source. Laser light is injected from a single-mode fiber into an integrated waveguide, which is evanescently coupled to the microdisk cavity of the optomechanical AFM probe. The transmitted light is coupled from the waveguide output into an output fiber and detected by a photodetector connected to a preamplifier, a lock-in amplifier, and an oscilloscope. The device is measured in air at room temperature. The cantilever is driven by the stochastic Langevin force from the thermal bath and an electrostatic force applied from a metal tip in close proximity to the cantilever.

**Fig. 2:**
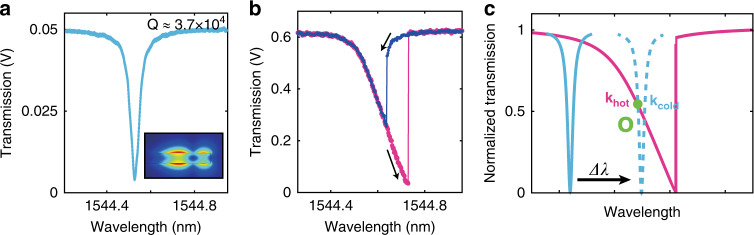
Whispering-gallery mode and thermo-optical nonlinearity. Linear and thermo-optically nonlinear transmission spectra of a WGM are shown in **a** and **b** for low and high input optical power, respectively. The radial-cross-sectional mode shape, i.e., the distribution of the electric field intensity, is shown in the inset. The hysteresis in **b** is obtained by changing the direction of the laser wavelength sweep, as labeled by the black arrows. *Q* ≈ 3.7 × 10^4^. **c** Schematic of the thermo-optical nonlinearity. The thermally induced refractive index change tunes the resonance frequency during the wavelength sweep (from the solid blue line to the dashed blue line), making it intensity-dependent, shown as the purple line. The absolute values of the slopes for the normalized linear and nonlinear spectra at a working wavelength *O* are *k*_cold_ and *k*_hot_, which are proportional to their corresponding transduction gains.

Figure 2a, b shows a WGM resonance dip in the transmission spectrum of the device under low and high-input powers, respectively. With increasing light intensity, the optical response exhibits the nonlinear effect evident from the hysteresis shown in Fig. 2b. It is noteworthy that the slope of the high-power transmission dip is linear, suggesting that the resonance spectral shift from thermo-optical tuning is linearly proportional to the power dropped into the microdisk, as discussed in detail later.

The broadening, asymmetry, and hysteresis in the transmission dip in these small Si cavities are generally attributed to the linear and nonlinear two-photon optical absorption in conjunction with the thermo-optical effect^16^ rather than the optical nonlinearity^21^. The small mode volumes and high *Q* of the cavities enhance the light-matter interaction, enabling sensitive measurement of the high-mechanical-bandwidth nanoscale cantilever probes, but also increase the local cavity heating and thermo-optical tuning. Miniaturization of the mechanical probes extends their useful mechanical transduction bandwidth from DC up to at least 10 MHz, a range that includes timescales both well below and high above the typical few-microsecond thermal time constants of nanophotonic cavities^22^, therefore, requiring accurate quantification of the thermal effects on signal transduction across the frequency range.

### Transfer function in the thermo-optically nonlinear regime

Temperature changes shift the cavity resonances via two main mechanisms: thermal expansion of the disk and the refractive index change of the cavity materials. For a small change in temperature Δ*T*_O,λ_, the relative wavelength shift can be written as:

1 $$\frac{{{\Delta}\lambda }}{{\lambda _0}} = \left( {\chi + \frac{1}{{n_0}}\frac{{{\mathrm{d}}n}}{{{\mathrm{d}}T}}} \right){\mathrm{{\Delta}}}T_{{\mathrm{O}},\lambda }$$

where *χ* = 2.6 × 10^−6^ K^−1^ is the thermal expansion coefficient of silicon, *n*_0_ ≈ 3.48 is the refractive index of silicon at the working wavelength *λ*_0_ ≈ 1.5 μm at room temperature, $\frac{{{\mathrm{d}}n}}{{{\mathrm{d}}T}} = 1.72 \times 10^{ - 4}\,{\mathrm{K}}^{ - 1}$ is the temperature sensitivity of the refractive index^23^ and Δ*T*_O,λ_ is the temperature change induced by the absorbed optical power. Equation (1) can be rewritten as $\frac{{{\Delta}\lambda }}{{\lambda _0}} = \alpha {\Delta}T_{{\mathrm{O}},\lambda }$, where, specifically for Si, $\alpha \approx \frac{1}{{n_0}}\frac{{{\mathrm{d}}n}}{{{\mathrm{d}}T}} = 4.94 \times 10^{ - 5}\,K^{ - 1} \gg \chi$. During the sweep of the laser wavelength across a WGM resonance, the cavity temperature increases linearly with the absorbed power. As shown in Fig. 2c, the rising temperature continuously shifts the optical resonance (blue dashed line) to longer wavelengths, generating a nonlinear optical transmission dip in the spectrum (purple line). At a working point *O* on the spectrum (determined by the chosen laser wavelength), we define the absolute value of the slope of the normalized “hot cavity” transmission dip as *k*_hot_ and the considerably steeper slope of the thermally shifted linear optical response (cold cavity) as *k*_cold_. Note that *k*_hot_ and *k*_cold_ are slopes of normalized optical modes scaled by the input optical power [cf. Supplementary Fig. S5 shows the slopes before scaling]. The working point *O* can be any point on the nonlinear resonance; however, the largest transduction gain is achieved for the wavelengths within the linear slope of the transmission dip’s shoulder.

Typical photonic cavities have short photon lifetimes, responding near-instantaneously to mechanical motion in the absence of heating effects. The thermo-optical effect complicates the optomechanical transfer function, coupling it to the thermal dynamics of the nanophotonic cavity. For a given microdisk incident intensity *I*_*i*_ (background intensity at off-resonance wavelengths) and a transmitted intensity *I* < *I*_*i*_ at *O*, the small transmitted intensity change Δ*I*_O,λ_ near the working point *O* of wavelength *λ* is a linear function of both the displacement Δ*x*_O,λ_ and the temperature change Δ*T*_O,λ_:

2 $$\frac{{{\Delta}I_{{\mathrm{O}},\lambda }\left[ {x,{\Delta}T_{{\mathrm{O}},\lambda }\left( I \right)} \right]}}{{I_i}} = k_{{\mathrm{cold}}}\left( {g_{{\mathrm{om}},\lambda }{\Delta}x_{{\mathrm{O}},\lambda } + \lambda _0\alpha {\Delta}T_{{\mathrm{O}},\lambda }} \right)$$

where $g_{{\mathrm{om}},\lambda } = \frac{{{\mathrm{d}}\lambda }}{{{\mathrm{d}}x}}$ is the optomechanical coupling coefficient for the cavity wavelength. For our photonic AFM probes, the typical *g*_om,λ_ ≈ 0.5 GHz/nm. In turn, the temperature *T* is a function of the intensity lost into the cavity *I*_*I*_ − *I*. The small temperature variation Δ*T*_O,λ_ near *O* can be obtained by considering the thermodynamic equation for the optical cavity with a thermal time constant *τ*:

3 $$\frac{{d({\mathrm{{\Delta}}}T_{{\mathrm{O}},\lambda })}}{{{\mathrm{d}}t}} = - \frac{1}{\tau }\left( {{\mathrm{{\Delta}}}T_{{\mathrm{O}},\lambda } + \eta {\mathrm{{\Delta}}}I_{{\mathrm{O}},\lambda }} \right)$$

where *η* is the proportionality constant between the temperature change Δ*T*_O,λ_ and the transmitted intensity change Δ*I*_O,λ_, whereby decreased transmitted intensity corresponds to increased heating. *η* is proportional to the product of the thermal impedance of the sensor and the ratio of the energy absorption to the total energy loss in the cavity (which also includes radiative leakage and scattering loss).

The complete frequency-dependent transfer function of the linearized system (2), (3) can be obtained by considering the system’s response at the working point *O* to a small harmonic cantilever motion, which perturbs the cavity resonance via optomechanical coupling. The optomechanically induced periodic resonance frequency shift modulates the power entering the cavity, resulting in both a small harmonic temperature variation Δ*T*_*O,λ*_ and the transmitter intensity variation Δ*I*_O,λ_:

4 $$\begin{array}{*{20}{c}} {{\Delta}x_{{\mathrm{O}},\lambda } = x_0e^{i\omega t}} \\ {{\Delta}T_{{\mathrm{O}},\lambda } = T_0e^{i\omega t}} \\ {{\mathrm{{\Delta}}}I_{{\mathrm{O}},\lambda }/I_i = i_0e^{i\omega t}} \end{array}$$

where the harmonic displacement of the cantilever has an amplitude *x*_0_ and frequency *ω*, *T*_0_ is the amplitude of the harmonic temperature variation and *i*_0_ is the relative intensity modulation amplitude. By combining Eq. (4) with Eqs. (2), (3), we obtain the small-signal intensity modulation, *i*_0_, response as:

5 $$i_0 = {\mathrm{{\Lambda}}}\left( \omega \right)x_0$$

where $r = \frac{1}{{1 + k_{{\mathrm{cold}}}\lambda _0\alpha \eta I_i}}$, *β* = *k*_cold_ *g*_om,λ_, and ${\mathrm{{\Lambda}}}\left( \omega \right) = \left( {1 - \frac{{1 - r}}{{i\omega \tau r + 1}}} \right)\beta$ is the complete frequency-dependent transfer function connecting the input signal amplitude *x*_0_ with the resulting relative intensity modulation *i*_0_. When either the incident intensity *I*_*i*_ is low or the intensity change is not converted into a temperature change (*η* ≈ 0), we have *r* ≈ 1, the microdisk temperature change Δ*T*_*O,λ*_ ≈ 0, and Δ*I*_O,λ_/*I*_*i*_ = *β*Δ*x*_O,λ_ is linear, with a constant transfer coefficient *β*. With increasing incident intensity *I*_*i*_, the ratio *r* decreases below unity, and the transfer function acquires the correction coefficient $\left( {1 - \frac{{1 - r}}{{i\omega \tau r + 1}}} \right) < 1$ arising from thermal dynamics and the thermo-optical effect. The magnitude of the transmission modulation is

6 $$\left| {i_0} \right| = \left| {\mathrm{{\Lambda}}} \right|x_0 = \beta x_0r\sqrt {\frac{{1 + \tau ^2\omega ^2}}{{1 + \tau ^2\omega ^2r^2}}}$$

**Fig. 3:**
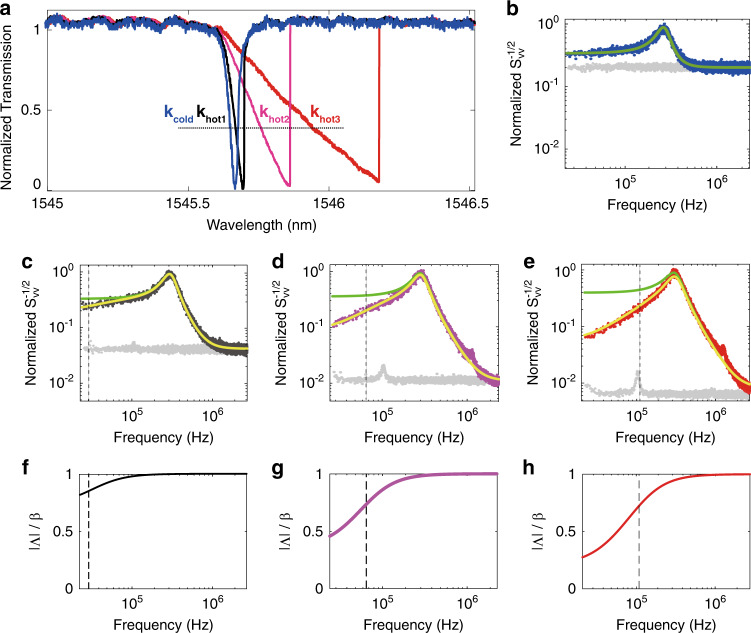
Optothermal-induced changes in the transfer function. **a** Normalized transmission signal with slopes of *k*_cold_, *k*_hot1_, *k*_hot2_, and *k*_hot3_. The cross points between the horizontal dashed line and the transmission signal mark the working wavelengths for **b**–**e**. **b**–**e** Normalized power spectral density of the optomechanically detected single-clamped cantilever thermal-mechanical fluctuations. They correspond to the normalized transmission data of the same colors shown in **a**. The green lines are the Lorentzian fit plus the measured background noise (gray dots), and the yellow lines are given by the nonlinear optical transfer function with no adjustable parameters needed. **f**–**h** The transfer function in **c**–**e** normalized by *β*. The vertical dashed lines mark 1/(2*πrτ*). *r*_1,2,3_ = *k*_*hot*1,2,3_/*k*_*cold*_ ≈ 0.74, 0.36, and 0.21. The low-input-power case (**b**) corresponds to a linear transmission dip; therefore, it has a normalized transfer function of unity.

Physically, the transfer function is equivalent to a high-pass filter, which attenuates the transduction gain of the sensor in the adiabatic frequency limit, *ω* ≪ 1/*τ*, by a factor of *r*: |*i*_0_| = *rβx*_0_. At high frequencies *ω* > 1/*τ*, the thermal dynamics do not play a role, |*i*_0_| = *βx*_0_, since the temperature change cannot follow the quick intensity modulation. By comparing the transfer function at the adiabatic limit and high-frequency limit, we see that the attenuation factor *r* describes the ratio of hot and cold cavity slopes, $r = \frac{{k_{{\mathrm{hot}}}}}{{k_{{\mathrm{cold}}}}}$. Experimentally, *r* is obtained directly from the ratio of the slopes of the measured normalized transmission spectra (Fig. 3a). The thermal time constant *τ* can be characterized experimentally by a number of common methods, including from time-domain or frequency-domain responses to a stimulus (e.g., optical power or frequency change) or by solving the heat equations via a finite-element method.

Figure 3a shows the normalized transmission signal of the optical mode. The scanning speed of the laser is ≈6 nm/s, which guarantees that the spectrum is taken in the adiabatic limit. With increasing input optical powers *P*_in_ (∝ *I*_*i*_), the dip in the spectrum becomes increasingly asymmetric due to the thermo-optical nonlinearity, and the slope decreases from *k*_cold_ to *k*_hot1_, *k*_hot2_ and *k*_hot3_, from which we obtain the ratios $r_{1,2,3} = \frac{{k_{{\mathrm{hot}}1,2,3}}}{{k_{{\mathrm{cold}}}}}$.

The thermally driven resonator is an ideal platform to demonstrate the transfer function since the Langevin force is frequency-independent (white) and simultaneously excites equally at all frequencies. Although the origin of the white fluctuations shown in Fig. 3 is irrelevant to the validity of this work and the most important data are obtained in the driven case shown in the next section, here, we attribute the white force noise driving the probe to the thermodynamic Langevin force, arising in accordance with the fluctuation-dissipation theorem and originating mostly from the ambient air, the leading source of damping in our system. Notably, thermal noise is used as one of the standard, well-established procedures for AFM cantilever calibration^12^. To ensure that the mechanical fluctuation signal is well above the detection noise floor, we cleaved the device to obtain a softer, single-clamped cantilever. It lowers the mechanical stiffness to ≈32 mN/m and the fundamental in-plane mechanical mode frequency to ≈310 kHz.

Figure 3b presents the normalized measured power spectral density as a function of the frequency $f = \frac{\omega }{{2\pi }}$ for the linear optical mode shown as the blue dip in Fig. 3a. The green line shows the corresponding Lorentzian fit (*Q* ≈ 3.0 due to air damping) plus the independently measured detection noise shown as the gray dots. Figure 3c–e presents the normalized power spectral density as the laser intensity is increased, corresponding to the transmission spectra of the same color shown in Fig. 3a. Larger spectral shifts are achieved (1 > *r*_1_ > *r*_2_ > *r*_3_). The green lines are the corresponding Lorentzian fit performed at *f* close to the peaks and larger than 1/(2*πrτ*) (vertical dashed lines), where the thermo-optical-nonlinearity-induced attenuation is small. Separately measured detection noise (gray dots) is added to the Lorentzian fit. The peak on the background at ~0.1 MHz is attributed to the vibration of floating parts of the waveguide. Note that because the thermal response has the same magnitude despite different transduction gains, the normalized detection noise floor becomes lower with increasing transduction gain, meaning a better detection sensitivity [gray dots in Fig. 3a–e]. At *f* ≫ 1/(2*πrτ*), the measured power spectral density agrees well with the Lorentzian fit. The deviation appears at *f* < 1/(2*πrτ*), where the thermo-optical response becomes significant.

Figure 3f–h shows the corresponding transfer function magnitude |Λ| normalized by *β*. This normalized optomechanical transduction coefficient is approximately $\frac{{\left| {\mathrm{{\Lambda}}} \right|}}{\beta } \approx 1$ for *f* ≫ 1/(2*πrτ*) and monotonically decreases with decreasing frequency, tending to $\frac{{\left| {\mathrm{{\Lambda}}} \right|}}{\beta } \approx r$ at *f* ≪ 1/(2*πτ*). Based on Eq. (6), the yellow lines in Fig. 3c–e are generated by multiplying the Lorentzian fits *β x*_0_(*ω*) (green lines) by the normalized transfer function |Λ|/*β*. *r*_1,2,3_ are obtained independently from the slopes in Fig. 3a. For our microdisk cavity, we used *τ* ≈ 7.0 µs, experimentally obtained by measuring the thermo-optical tuning signal when intensity-modulated input power is applied^24^. A finite-element method numerical modeling of the thermal dynamics of the system also gives the same value (Supplementary Materials). Therefore, the transfer function does not include any adjustable parameters or any numerical simulations, while a good agreement is achieved for all powers. As another choice, *τ* can also be obtained as the only fitting parameter in the transfer function. Fitting to the data in Fig. 3c–e gives *τ* = (6.7 ± 0.4) μs.

One consequence of thermo-optical tuning is that the transduction gain at low frequency will saturate with increasing input power. In the linear regime, the transduction gain is proportional to the input optical power ∝*P*_in_ ∝ *I*_*i*_, i.e., the same amount of resonance shift induces a larger transmitted power variation for a higher *I*_*i*_. However, the transfer function is attenuated by a factor of *r* at the adiabatic limit [*f* ≪ 1/(2*πτ*)] in the nonlinear regime, saturating the transduction gain ∝ *rI*_*i*_ with increasing input power as $rI_i = \frac{{I_i}}{{1 + k_{{\mathrm{cold}}}\lambda _0\alpha \eta I_i}} \approx \frac{{I_i}}{{k_{{\mathrm{cold}}}\lambda _0\alpha \eta I_i}} \propto \frac{{I_i}}{{I_i}}$ when *r* ≪ 1 (Supplementary Materials). Note that saturation occurs only in the low-frequency region and that the linear dependence of the gain on *I*_*i*_ at high frequency [*f* ≫ 1/(2*πτ*)] is still valid in the nonlinear regime. Therefore, with increasing input power, the system continuously passes three different regimes, namely, the linear regime, nonlinear regime, and saturation regime defined by ∆*λ*, being much smaller, comparable, or much larger than the linewidth of the optical mode, respectively.

### Improvement in displacement sensitivity achieved by operating in the nonlinear regime

The derived high-incident-power transfer function in the thermo-optically nonlinear regime is easily invertible and enables increased sensitivity in broadband measurements of probe displacement. To demonstrate the sensitivity increase, a probe response to an external drive force is measured in the high-power, nonlinear regime and compared to the best measurement in the linear regime. The measurement is performed on a regular probe with a double-clamped cantilever that we used in previous experiments^14,15^ (electron micrograph in Fig. 1a). The cantilever is driven by an electrostatic force applied via a metal tip in close proximity to the cantilever (Fig. 1c). The optical transmission carrying the displacement signal is demodulated at the frequency of the drive by a lock-in amplifier.

**Fig. 4:**
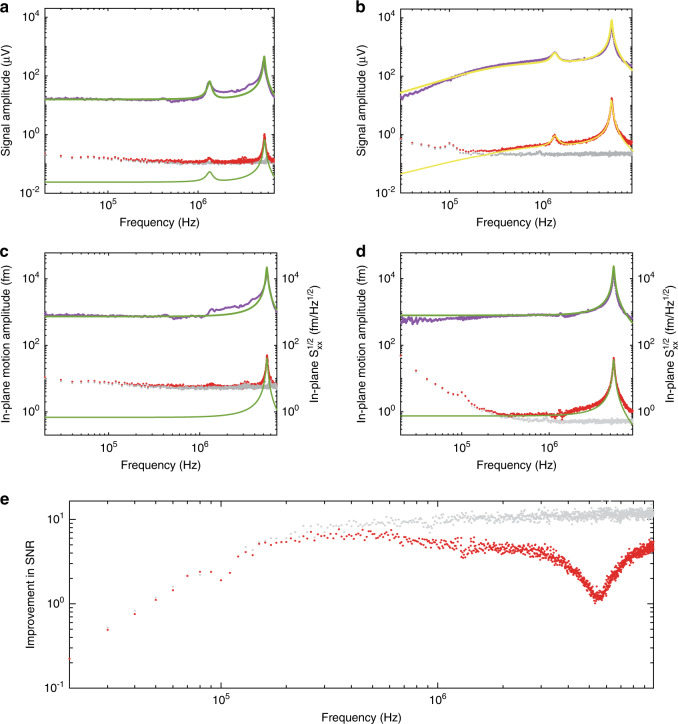
Sensitivity improvement in the thermo-optical regime. Electrostatically driven response (purple), thermally driven response plus detection noise (red), and detection noise alone (gray) are measured in the linear and nonlinear regimes, shown in **a** and **b**, respectively. The noise amplitudes are within a 1 Hz equivalent bandwidth. Green lines are the corresponding Lorentzian fit. Yellow lines are Lorentzian fits multiplied by the transfer function, accounting for the thermo-optical nonlinearity. **c**, **d** The calibrated in-plane motion amplitudes from **a** and **b**, respectively, where the out-of-plane signals are removed. **e** Signal-to-noise ratio improvement for force sensing limited by thermal noise (red) and for the displacement sensing limited by detection noise (gray).

Figure 4a shows the best achievable performance of the AFM probe in the linear regime where the input optical power is set at the threshold where the nonlinearity is about to appear. Figure 4b shows the data measured with the same drive strength but with increased optical power in the nonlinear regime, where a much stronger sensor signal is evident for the same driven motion. The electrostatically driven response (purple dots) is measured while scanning the driving frequency. The measurement noise, shown as the red dots, is recorded while the drive is off and consists of the probe’s thermal-mechanical noise added to the detection noise background (gray dots). The measured noise voltage is plotted with a 1 Hz equivalent noise bandwidth. The dominant detection noise in the system is typically either detector dark noise for low detected power or optical shot noise for high detected power (Supplementary Materials), while at lower frequencies, the laser frequency and amplitude technical noises may become significant as well, depending on the optical power and the instrument chosen.

The Lorentzian fits (green lines) to the driven mechanical signal show the fundamental in-plane mode with a resonance frequency of ≈5.46 MHz, quality factor of ≈31.4, and a much weaker signal originating from the out-of-plane mode at ≈1.35 MHz of a ≈9.8 quality factor. The AFM probe is designed to be used such that the measured physical quantities, such as the topography and thermal properties of the substrate, generate mostly in-plane motion^14,15^ while the tip is in contact with a sample surface oriented normal to the chip plane^15^. Therefore, here, we focus only on the in-plane mode.

In the linear regime in Fig. 4a, the noise from thermal-mechanical fluctuations is largely below the detection noise, except for the frequency around the fundamental in-plane mode resonance. Because of the higher transduction gain with higher optical power in Fig. 4b, the thermal noise is amplified above the detector background over most of the frequency range. However, for both the driven motion signal and the thermal noise the increase of transduction gain is suppressed at low frequencies by the attenuation factor *r*, resulting in the gain saturation. The readout data are quantitatively described by the transfer function considering the thermo-optical nonlinearity (yellow line) where *r* ≈ 0.05 is independently obtained from the optical spectrum and *τ* ≈ 7.0 μs from the numerical simulation is used.

Using the equipartition theorem and the measured thermal noise data, we calibrate the sensitivity to the in-plane displacement as ≈0.021 and ≈0.36 V/nm (at high frequency) for the linear and nonlinear cases, respectively (Supplementary Materials). We exclude the out-of-plane signal by subtracting the Lorentzian fit of the out-of-plane mode signal. The readout gain in the linear regime is frequency-independent, and the measured voltage is directly converted to displacement, as shown in Fig. 4c. The frequency-dependent gain in the nonlinear regime is given by the transfer function, and the displacement is shown in Fig. 4d. The increased transduction gain effectively improves the noise level of the optomechanical sensor from the best performance in the linear regime of ≈6 fm/Hz^1/2^ to ≈0.7 fm/Hz^1/2^ at frequencies out of resonance, a ≈10-fold improvement. In contact mode operation, the probe tip touches the sample surface, and the thermal noise is strongly suppressed^14,15^ due to the increased stiffness from the mechanical contact with the sample. Therefore, further optomechanical gain increases and signal-to-noise ratio (SNR) improvement would be possible before thermal-mechanical noise becomes the dominant noise source, i.e., the red dots in Fig. 4e would move up, and the gray detection limit SNR improvement can be raised even higher at high frequencies by a further optical power increase. Considering the detector background, the probe displacement is measured here with an uncertainty of ≈0.4 fm/Hz^1/2^, which is the performance expected in the contact mode. Figure 4e shows the improvement in the SNR, achieved by increasing the optical power, as a function of the frequency. The gray data show the improvement in performance for sensing the displacement of the probe, such as the sample topography, while the red data illustrate the performance improvement for sensing the force, where the motion fluctuations driven by the thermal Langevin force impose an additional limit. In the case of force sensing, the increase in the transduction gain in the nonlinear regime improves the performance of the sensor from detection-noise-limited to thermal-noise-limited, and the improvement ratio depends on the ratio of the detection noise to the thermal noise in the linear regime. For example, the SNR improvement ratio at the eigenfrequency of the fundamental in-plane mode is approximately 1 since the thermal response is already much larger than the detection noise at the resonance in the linear regime (Fig. 4a), while it is between 1 and 10 at the frequency out of resonance. Another pronounced feature is that the improvement is lower than 1 at *f* < 30 kHz. This is attributed to the saturation of the transfer function at low frequency and the increase in detection noise with the increase in input optical power due to both shot and technical noises. Because the gain increases linearly and the shot noise increases as the square root of the power, the displacement SNR always improves with the power for high mechanical frequencies.

### Improving the sensitivity of nanophotonic sensing by lowering the optical quality factor

Conventionally, a low-loss optical cavity with high *Q* is always desired for nanophotonic sensing since increasing the photon lifetime leads to longer interaction, higher transduction gain, and increased sensitivity. Additionally, as we present above, the sensitivity can be further improved by increasing the optical power. However, the combination of a high optical *Q* and a high optical power does not guarantee the best sensitivity, as intuitively expected, i.e., an optical cavity with a lower *Q* can have larger optimal (saturated) transduction gains and better sensing performance. Qualitatively, this counterintuitive conclusion is due to the fact that for the low-frequency input signals, the gains of high-*Q* optical modes saturate more quickly with increasing input power, while the gains of low-*Q* modes saturate later and possibly at higher gain values.

**Fig. 5:**
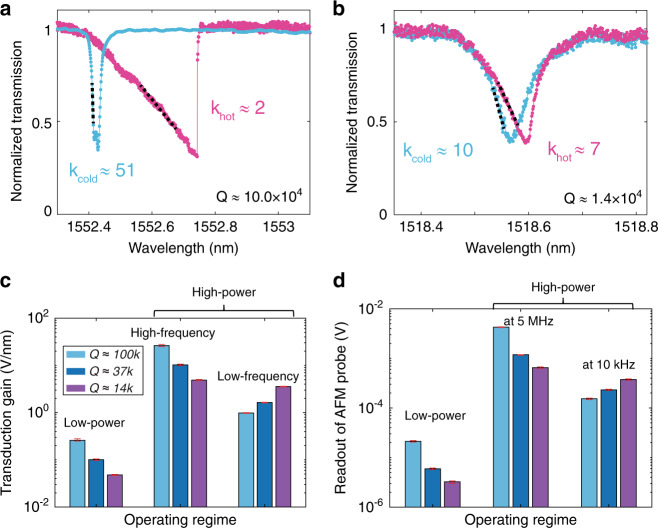
Transduction gain of nanophotonic modes with different quality factors. **a**, **b** are two measured optical modes from the first- and third-radial-order families of the microdisk, with very different *Q* ≈ 10.0 × 10^4^ and ≈1.4 × 10^4^, respectively. The blue line and purple line correspond to *I*_*i*_ ≈ 0.01 V and *I*_*i*_ ≈ 0.98 V, respectively. Their slopes at the working wavelength are indicated by the black dashed lines. **c** Transduction gains normalized by *g*_om,λ_. The light blue, dark blue, and purple bars correspond to modes of radial order from 1 to 3, respectively. The operating regimes from left to right correspond to the low-input-power regime (*I*_*i*_ ≈ 0.01 V), the high-input-power (*I*_*i*_ ≈ 0.98 V) and high-frequency regime, and the high-input-power and low-frequency regime, where the transduction gain saturates due to thermo-optical tuning. The low-frequency gain at saturation counterintuitively increases with decreasing *Q*. **d** Readout of the AFM probe at the corresponding regimes shown in **c**. The one-standard-deviation statistical uncertainties marked in **c** and **d** are obtained from the uncertainty of the linear fit for the transmission dip and the measured electrostatically driven response at the specific frequencies, respectively.

Figure 5a, b presents two measured microdisks WGMs with very different *Q* ≈ 10.0 × 10^4^ and ≈1.4 × 10^4^, respectively. When the input power is low, the blue lines show that the absolute value of the slope *k*_cold_ of the high-*Q* mode is higher, meaning a higher transduction gain, given an equal *g*_om,λ_. For the high input power, the purple lines show that the high-*Q* mode in Fig. 5a is fully nonlinear (the gain is saturated), while the lossy mode in Fig. 5b is still nearly linear (the gain is not saturated) under the same input power, and it has a higher *k*_hot_ at the working wavelength despite its lower *Q*. In other words, the optimal (saturated) transduction gain of the low-*Q* modes is shown to be better than that of the high-*Q* modes at low frequency.

The optomechanical transduction gains normalized by *g*_om,λ_ are shown in Fig. 5c, as determined by the slopes of the optical resonances. The modes of *Q* ≈ 10.0 × 10^4^, 3.7 × 10^4^, and 1.4 × 10^4^ are from the first- (Fig. 5a), second- (Fig. 2), and third-radial-order (Fig. 5b) families, and they are colored light blue, dark blue, and purple, from left to right. At low-input powers, where the modes are linear and Δ*T*_O,λ_ ≈ 0, the transduction gain $\frac{{{\mathrm{{\Delta}}}I_{{\mathrm{O}},\lambda }}}{{{\mathrm{{\Delta}}}x_{{\mathrm{O}},\lambda }}} = I_ig_{{\mathrm{om}},\lambda }k_{{\mathrm{cold}}}$ based on Eq. (2). Therefore, modes with higher *Q* provide greater *k*_cold_ and transduction gain, given equal values of *g*_om,λ_. For high input powers, at high frequencies [*f* ≫ 1/(2*πτ*)], the transfer function is not affected by thermal dynamics; therefore, the transduction gain has the same trend as the low-power regime. In contrast, the thermal dynamics at low-frequency results in gain saturation at high-input power [Supplementary Materials Note 2]. The high-*Q*, high-circulating-power optical modes convert more light into heat and saturate earlier, while the optical modes of lower *Q* saturate at higher input power, allowing them to achieve higher transduction gain. The saturated gain is proportional to $rI_i = \frac{1}{{k_{{\mathrm{cold}}}\lambda _0\alpha \eta }}$, where *λ*_0_ and *α* are constant for the modes under consideration. *η* is proportional to the ratio of the energy absorption loss to the total energy loss in the cavity, given by the ratio of their loss rates: $\eta \propto \frac{{\gamma _{\mathrm{a}}}}{{\gamma _{{\mathrm{total}}}}}$, while $k_{{\mathrm{cold}}} \propto \frac{1}{{\gamma _{{\mathrm{total}}}}}$, where *γ*_total_ is the total loss of the microdisk and *γ*_a_ is the absorption loss rate due to the energy being converted to heat. Therefore, the saturated gain at low frequency is $\propto \frac{{\gamma _{{\mathrm{total}}}^2}}{{\gamma _{\mathrm{a}}}}$. For nanophotonic resonators with multiple well-confined modes, the absorption is mostly a cavity material property and tends to vary little with the mode order^24,25^. Large differences in *Q* and *γ*_total_ arise mainly from the differences in the surface-scattering loss and radiative loss, which strongly depend on the mode field distribution. Specifically, for the nanophotonic cavity used in this paper, our recent dissipation analysis^24^ shows that *γ*_a_ is nearly the same for the multiple studied modes (10 modes with 3 different radial orders) despite the order-of-magnitude difference in *γ*_total_. Because *γ*_a_ is approximately constant, the saturated (maximum achievable) gain at low frequency increases rapidly with the increase in the total dissipation rate *γ*_total_, i.e., with decreasing quality factor *Q*.

We experimentally demonstrate the anomalous *Q*-dependence of the transduction signal on the AFM probe, as shown in Fig. 5d. We drive the cantilever of the AFM probe to generate a fixed mechanical response and measure the response using the three optical modes considered in Fig. 5c. The readout signal for the same mechanical response at high frequency (≈5 MHz) increases with the *Q* of the optical transduction modes. However, the readout at low frequency (≈10 kHz) is the opposite; i.e., at low frequencies, the low-*Q* optical mode has a higher optimal transduction gain than that of the high-*Q* mode. In the broad context of frequency-based sensors, ours is not the only observation of lower quality factors leading to better sensitivity, cf. recent mechanical sensing experiments^26^. However, the underlying mechanism reported here is different, and to the best of our knowledge, no systematic analysis of this anomalous *Q*-dependence of transduction gain has been reported previously in the context of the thermo-optical effect.

The novel relationships revealed here between the input power, quality factor, and transduction gain could provide a framework to guide the design strategy of optical sensors. For example, the low-frequency sensitivity can be improved by designing a radiatively lossy cavity and increasing the waveguide coupling when high input power is readily available. In contrast, measurement at high frequency or with limited input power available should be done using high-*Q* modes to increase the time each photon is kept in the cavity and interacts with the probe. Although the trade-off between these parameters should be discussed depending on the specific parameter range, decreasing the absorption loss rates and enhancing the heat sinking of the optical cavity are generally helpful to improve the sensitivity and the quality factors.

## Conclusions and outlook

In summary, we derived and experimentally validated a broadband, small-signal transfer function valid for the high optical power, thermo-optically nonlinear regime of photonic sensing. The transfer function uses the thermal time constant independently obtained from finite-element modeling and the gain ratios separately measured from optical spectra. Excellent agreement with the optomechanically transduced power spectral densities of a nanocantilever was obtained without adjustable parameters, enabling quantitative optomechanical motion transduction with high sensitivity over a large frequency bandwidth down to DC. The displacement measurement noise of our optomechanical AFM probe improved from the best achievable performance ≈6 fm/Hz^1/2^ in the linear regime to ≈0.7 fm/Hz^1/2^ in the thermo-optical nonlinear regime. Not limited to optomechanical transducers, this work demonstrated a general and practical approach for improving the sensitivity of a broad range of technologically important photonic resonator sensors subject to thermo-optical tuning via optical absorption.

Additionally, the interplay between the thermo-optical effect and the dissipations of the nanophotonic cavity revealed a new counterintuitive regime where the low-*Q* modes possess higher optimal transduction gain at low frequency. The new regime may inspire new strategies of design for nanophotonic sensors for low-frequency and static applications, such as topography scanning^27^ and sensing the dynamics of cells^28^.

## Materials and methods

### Numerical simulation

The numerical simulation of the thermal dynamics of the microdisk was performed by commercial finite-element-method software. The geometries used in the simulation included all relevant components, including the microdisk, supporting post, cantilever, frame, and substrate. Since the supporting post is the main thermal impedance, we carefully measured its dimensions on a testing sample released together with the device. The diameters of the top and bottom surfaces of the post defined by the hydrofluoric acid etching process were measured to be ≈1.5 and ≈2.2 µm, respectively. The whole setup was situated in around a one-standard-atmosphere and room-temperature environment.

In the simulation, the input power was applied at time zero to the rim of the microdisk, where the optical modes were located. By fitting the thermal response of the microdisk, we obtained its thermal constant *τ* ≈ 7.0 µs (Supplementary Materials).

## Supplementary information

Supplementary Materials
