# Supplementary material for: Overcoming thermo-optical dynamics in broadband nanophotonic sensing
Source: Microsyst Nanoeng. 2021 Jul 7;7:52. doi: 10.1038/s41378-021-00281-y (PMC8433424; doi:10.1038/s41378-021-00281-y)
Supplement: Supplementary file 1 — Supplementary Materials [file 41378_2021_281_MOESM1_ESM.docx]

**Supplementary Materials**

Overcoming Thermo-Optical Dynamics in Broadband Nanophotonic Sensing

Mingkang Wang^1,2,†^, Diego J. Perez-Morelo^1,2^, Vladimir Aksyuk^1, *^

^1^Microsystems and Nanotechnology Division, National Institute of Standards and Technology, Gaithersburg, MD 20899 USA

^2^Institute for Research in Electronics and Applied Physics, University of Maryland, College Park, MD 20742, USA

1. **Numerical simulation of the thermal effect**

As described in the main text, our nominally 10 µm diameter, 260 nm thick silicon microdisk is supported at the center by a 2 µm thick silica post. The diameter of the top and bottom surface of the post defined by the hydrofluoric acid etching process is measured to be ≈ 1.5 µm and ≈ 2.2 µm. The whole setup is in an air environment.

In the simulation, an input power of $P_{in}$ ≈ 0.1 mW is applied at time zero to the rim of the microdisk where the optical modes are located. Figure S1 (a) shows the temperature distribution of the system at the thermal equilibrium. The post is the main source of thermal impedance, while the Si substrate has high-enough thermal conductivity to be nearly isothermal. The effect of the cantilever on the simulation results is less than 2%, however, for the accuracy, we also included the cantilever in the model. The time-domain thermal simulation result is presented in Figure S1 (b). By fitting the curve with $T=T'+\Delta Te^{-\frac{t}{\tau}}$, we obtain the thermal response time $\tau$ ≈ 7.0 µs, and $\kappa$ ≈ 1.5 × 10^5^ K/W. $T^{'}=293 K$ is a constant offset and $\Delta T=15 K$.


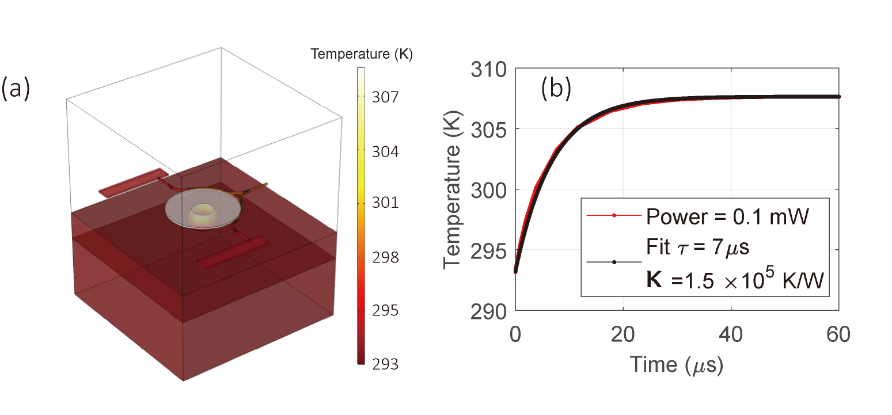


Figure S1. Simulated thermal response of the silicon microdisk. (a). Thermal distribution on the microdisk at equilibrium. (b). Dynamic response of the disk to the application of a known thermal load at the periphery.

1. **Transduction gain saturation**


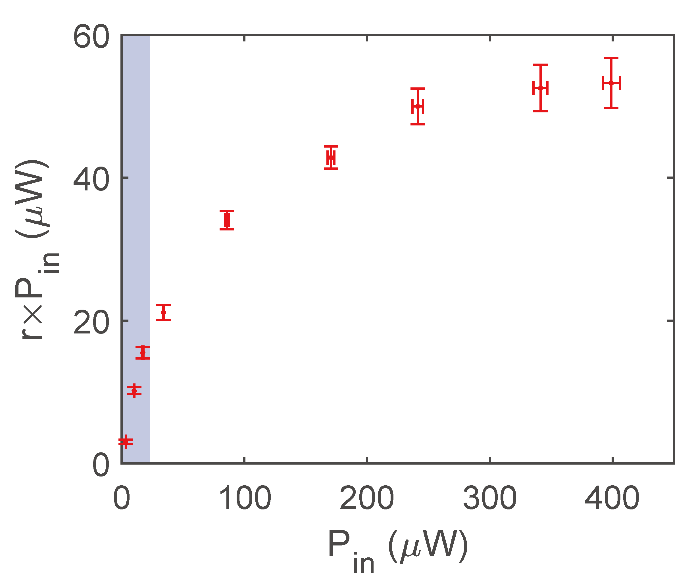


Figure S2. Product of the transfer function parameter $(r)$ and the input power ($P_{in})$ as a function of $P_{in}$. $rP_{in}$ approaches saturation as $r$ decreases with increasing $P_{in}$.

Figure S2 shows the measured ${rP}_{in}$ where $r=\frac{k_{hot}}{k_{cold}}$ is measured from the transmission signal at the corresponding $P_{in}$. It shows that the transduction gain, $\propto rP_{in}$, at zero-frequency tends to saturate for high input powers, becoming independent of the input power $P_{in}$.

Note, the saturation only occurs at zero-frequency in the nonlinear regime, the linear dependence of the gain on $P_{in}$ at high frequency is still valid.

1. **Probe response noise in the frequency domain and probe response equipartition calibration**

Figure S3 (a) and (b) show the measured noise power spectral densities of the AFM probe with a double-clamped cantilever in the nonlinear and linear regime, respectively. The dark noise (black) of the photodetector is measured when the input optical power is set to 0, the shot noise + dark noise is measured when the same optical power is transmitted at the working wavelength set at the background of the optical transmission spectrum (transduction gain = 0), and the thermal noise (red) is finally included when the working wavelength is tuned to the shoulder of the optical dip. It is noteworthy the total noise power spectral density (red) also contains the experiment-dependent technical noises such as optical fiber vibration and laser frequency instability, however, they mainly exist at low mechanical frequency range (<0.1 MHz).

We convert the voltage power spectral density to input-optical-power-dependent displacement noise [Fig. 4(a) (c)] by using the equipartition theorem. First, we modeled the device with a finite element method (FEM) which gives the correct mechanical eigenfrequencies, the same as the measuring device. Next, we obtain the effective spring constant of the fundamental in-plane mode as $k_{in}\approx2.03 N/m$. Second, the integrated voltage noise power [area under the green line in Fig. S3(a)] is equal to $\left\langle x_{in}^{2} \right\rangle=\frac{k_{B}T}{k_{in}}$ as the result of the equipartition theorem. It gives the calibration constant $a_{1}\approx2.8 fm/\mu V$ for (a). As the transduction gain depends on the input power, the calibration constant also depends on the input optical power. However, as the calibrated displacement does not change with gain, we can easily obtain the calibration constant for (b) as $a_{2}\approx48.1 fm/\mu V$. $a_{1}$ and $a_{2}$ are used in the calibration of Fig. 4(c) and (a), respectively.


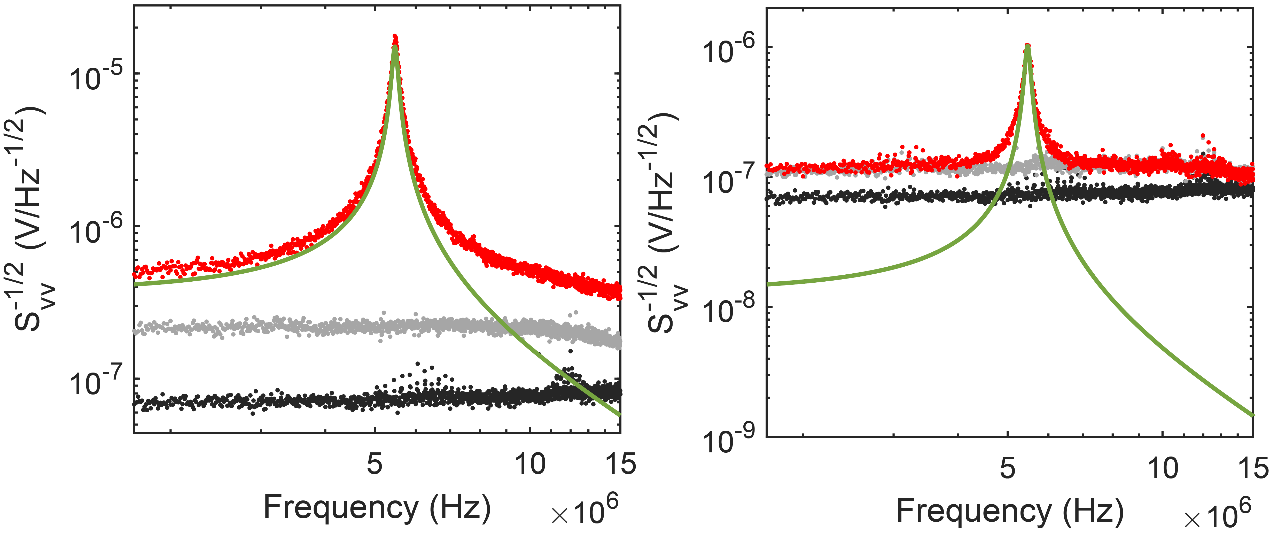


Figure S3. Power spectral density of dark noise (black), dark noise+shot noise (gray), dark noise+shot noise+thermal noise (red), and the Lorentzian fit to the thermal noise only (green) in nonlinear case (a) and linear case (b).

1. **Detection noise at the low frequencies (*f* << 1/2πτ)**

In the present experimental apparatus, the detection noises such as 1/*f* electronic noise and low-frequency mechanical vibration noise become non-negligible as frequency goes low. These noise sources are not fundamental and are expected to improve with a lower frequency noise, more stable laser, and better detection electronics. As shown in Fig. S4, combined noise (red dots) is dominated by the detection noise (gray dots) at a frequency below 0.1 MHz. Note, in order to show the spectrum at low frequency, we reduced the averaging of the Fast Fourier Transform (FFT), leading to a noisier spectrum compared to those shown in the main text. The driven signal obtained in the thermo-optical nonlinear regime (purple) is overwhelmed by the detection noises at frequencies < 10 kHz. By further increasing the input optical power, the transduction gain of high-frequency (>1 MHz) and intermediate-frequency (10 kHz – 1 MHz) signal can be further increased, while the low-frequency (<10 kHz) gain will only increase until it saturates, as shown in Fig. S2. Importantly, as we discussed in the section about anomalous Q-dependence of transduction gain, reducing the quality factor of the optical resonator could improve the saturated gain at low frequency, making it possible to get signal over the detection noise, i.e. the yellow line will be higher than the noise floor shown as the purple dots at frequencies lower than 10 kHz. Interestingly, low-quality factor devices under strong optical input could show better performance in a broadband measurement.


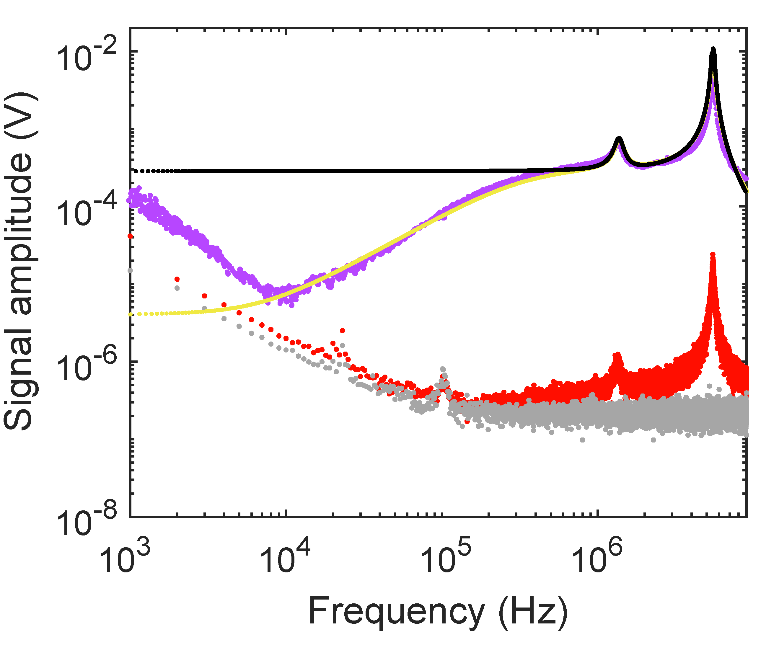


Figure S4. Power spectral density of detection noise (gray), detection noise and thermal noise (red), the driven response in nonlinear regime (purple), and the Lorentzian fit with (yellow)/without (black) considering the derived transfer function.

1. **Transmission signal before normalization**


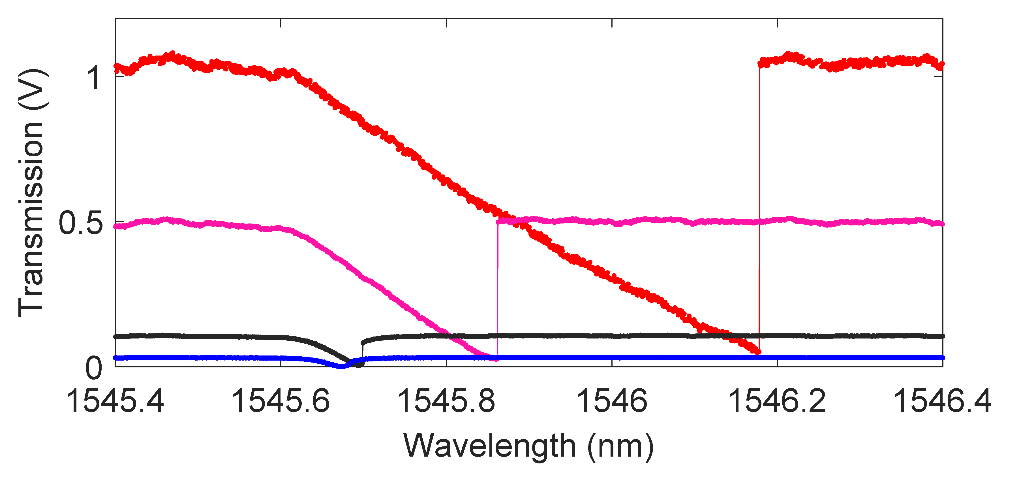


Figure S5. Transmission signal before normalization. The slopes of the optical modes are proportional to the transduction gain. For better presentation, we normalized transmission in the main text, where k_hot_ and k_cold_ are defined on the normalized modes.
